# Prevalence of Access to Prenatal Care in the First Trimester of Pregnancy Among Black Women Compared to Other Races/Ethnicities: A Systematic Review and Meta-Analysis

**DOI:** 10.3389/phrs.2022.1604400

**Published:** 2022-07-04

**Authors:** Pedro Henrique Alcântara da Silva, Kezauyn Miranda Aiquoc, Aryelly Dayane da Silva Nunes, Wilton Rodrigues Medeiros, Talita Araujo de Souza, Javier Jerez-Roig, Isabelle Ribeiro Barbosa

**Affiliations:** ^1^ Postgraduate Program in Public Health, Federal University of Rio Grande do Norte, Natal, Brazil; ^2^ Faculty of Health Sciences and Welfare, University of Vic–Central University of Catalonia, Barcelona, Spain

**Keywords:** systematic review, prenatal care, access to health services, racial factors, black women

## Abstract

**Objective:** To analyze the prevalence of access to prenatal care in the first trimester of pregnancy among black women compared to other races/ethnicities through a systematic review and meta-analysis.

**Methods:** Searches were carried out at PUBMED, LILACS, Web of Science, Scopus, CINAHL, and in the grey literature. The quality of the studies and the risk of bias were analyzed using the Joanna Briggs Critical Appraisal Checklist for Analytical Cross-Sectional Studies instrument. The extracted data were tabulatesd and analyzed qualitatively and quantitatively through meta-analysis.

**Results:** Black women had the lowest prevalence of access to prenatal services in the first trimester, with prevalence ranging from 8.1% to 74.81%, while among white women it varied from 44.9 to 94.0%; 60.7% of black women started prenatal care in the first trimester, while 72.9% of white women did so.

**Conclusion:** Black women compared to other racial groups had lower prevalence of access to prenatal care, with less chance of access in the first trimester, and it can be inferred that the issue of race/skin color is an important determinant in obtaining obstetric care.

**Systematic Review Registration:** https://www.crd.york.ac.uk/prospero/display_record.php?ID=CRD42020159968_, PROSPERO CRD42020159968.

## Introduction

Access to prenatal care is considered one of the main prognostic indicators for a healthy birth, contributing to a delivery with less impact on maternal health. The beginning of prenatal care should occur in the first trimester, as it stratifies pregnancy risk, achieving the appropriate number of consultations, and the performance of the recommended procedures and adequacy definers, avoiding unfavorable obstetric and neonatal outcomes, which may contribute to the reduction of maternal morbidity and mortality [1].

Prenatal care is the period before birth and it is composed of clinical and educational procedures with the goal of monitoring the evolution of the pregnancy. The Brazilian Ministry of Health recommends that prenatal care should start in the first trimester of pregnancy, as it makes it possible to reach the appropriate number of consultations, at least 6 overall, as well as to carry out the recommended procedures that define its suitability such as blood screening tests and fetal monitoring.

People of African descent, in addition to having limited access to health services, quality, and safe housing, also suffer from various discrimination, especially regarding class, race, and gender [1]. These aspects are evident in people of black race/skin color from all over the world. Which has repercussions on the life and death of these individuals [2]. When comparing women of white race/skin color, the black women, the interaction between biological, social, and environmental factors makes them more vulnerable to some diseases such as gestational hypertension and gestational diabetes mellitus [3].

Maternal mortality or pregnancy-related mortality provides one of the most striking examples of disparities in women’s health. Currently, the global maternal mortality ratio is around 210 deaths per 100,000 live births. According to Martins (2006), the Center for Disease Control and Prevention reports a decrease in the coefficients of maternal death in the United States from 319.8 to 5.7/100,000 live births among women white and from 781.7 to 18.6/100 thousand live births among black women, in the period 1940 to 1990. This 2 to 4 times greater ratio for black women is explained by the large number of pregnancy with morbidity, difficulty in accessing and using health services, in addition to the quality of care provided, or care received.

Pachecho (2018), conducted a study in the United Kingdom, identifying that 76,158 pregnant women found the risk of fetal death two times higher in black pregnant women compared to white pregnant women, in addition to significantly higher in all adverse outcomes (low birth weight, preeclampsia, preterm, pregnancy-specific hypertension and gestational diabetes mellitus), except elective cesarean section. There are persistent racial disparities in perinatal outcomes, with neonatal mortality rates in black women twice that of white women and maternal mortality rates in black women three to four times that of white women [4].

Thus, identifying whether the issue of race/skin color directly influences access to maternal health service with studies with greater scientific evidence is necessary, as it can elucidate a racial inequity with fatal consequences [5]. This debate may allow the development of public policies of racial equity in maternal health services with greater technical and scientific support, aiming at improving the quality of care in obstetrics and perineonatology.

Thus, this study aims to analyze the prevalence of access to prenatal care in the first trimester of pregnancy among black women compared to other races/ethnicities in a systematic review and meta-analysis and to identify the magnitude of the association.

## Methods

For the writing of this systematic review, we used the guidelines of the PRISMA Checklist (Main Items for Reporting Systematic Reviews and Meta-analyzes) [6]. The initial protocol for this review was registered on the PROSPERO platform under the number CRD42020159968.

In this study, the focused research question was: “What is the prevalence of access to prenatal care in the first trimester of pregnancy in black women compared to other races/ethnicities?” The inclusion and exclusion criteria of the articles obtained during searches in the databases were developed based on the PICO strategy [7] Participants–pregnant women, including adolescents, without nationality restriction at any gestational age; Exposure–black pregnant women; Controls–pregnant women from other ethnic or racial groups; Outcome–access to prenatal care in the first trimester; Type of studies–Cross-sectional studies. Cohort, case-control studies and literature reviews (integrative, narrative, or systematic with or without meta-analysis), case series, case reports, and qualitative studies were not considered.

On 10 October 2019, with a subsequent update on 19 February 2021, the following databases were accessed electronically: LILACS, PubMed, Scopus, Web of Science, and CINAHL, in addition to Google Scholar and the Opengrey.org portal to identify files stored in the grey literature (the gray literature includes dissertations, theses, official government reports), without restriction as to the language of the articles or date of publication. The search strategy combined the Mesh terms for “pregnancy”, “prenatal care” and “Health Services Accessibility”, in addition to related free terms (Table 1). Terms related to race, ethnicity, or skin color were not included, since the combination of these terms did not result in findings relevant to the objectives of this study.

**TABLE 1:** Search strategy (Brazil, 2021).

(“Pregnancy” or “Gestation”) and (“Prenatal Care” or “Antenatal Care”) and (“Health Services Accessibility” or “Access to Health Care”)	
**Strategy Database**	**Database**
LILACS	(“Pregnancy” or “Gestation”) and (“Prenatal Care” or “Antenatal Care”)” or “Prenatal Care” or “Antenatal Care” and (“Access to Health Care” or “Health Services Accessibility”)
PubMed	(“Pregnancy” [Mesh] or “Gestation”) and (“Prenatal Care” [Mesh] or “Antenatal Care”) and (“Health Services Accessibility” [Mesh] or “Access to Health Care”)
Scopus	(“Pregnancy” or “Gestation”) and (“Prenatal Care” or “Antenatal Care”) and (“Health Services Accessibility” or “Access to Health Care”)
Web of Science	(“Pregnancy” or “Gestation”) and (“Prenatal Care” or “Antenatal Care”) and (“Health Services Accessibility” or “Access to Health Care”)
CINAHL	(“Pregnancy” or “Gestation”) and (“Prenatal Care” or “Antenatal Care”) and (“Health Services Accessibility” or “Access to Health Care”)
Google Scholar	(“Pregnancy” or “Gestation”) and (“Prenatal Care” or “Antenatal Care”) and (“Health Services Accessibility” or “Access to Health Care”)
OpenGrey	(“Pregnancy” or “Gestation”) and (“Prenatal Care” or “Antenatal Care”) and (“Health Services Accessibility” or “Access to Health Care”)

Initially, the results obtained in the databases were inserted in the reference manager Mendeley Desktop^®^ version 1.19.4 for the elimination of duplicate studies. Then, the resulting articles were exported to Rayyan QCRI^®^ software to be screened by reading the title and abstract, applying the eligibility criteria. Even using the same software, after the initial screening, the studies selected in the previous step were submitted to full-textual reading to observe the completeness of the articles in the inclusion criteria. From this analysis, studies were obtained to be included in this systematic review and whose data were extracted.

In all stages of study selection, two reviewers trained to select studies, to use analysis software, and to extract data worked independently. To ensure the quality of the process, the divergences were solved by consensus. In cases where the disagreements persisted, a third reviewer was asked to solve the conflicts. The bibliographic references listed in the included studies were also analyzed to identify articles that met the inclusion criteria.

Two reviewers independently extracted study data and discrepancies at this stage were resolved by discussion and consensus. The following information from the observational studies was extracted and presented in a table: 1) Reference of the article (authors, place, year of publication); 2) Sample size; 3) Sociodemographic characteristics (race/ethnicity; the mean age of participants); 4) Prevalence of prenatal consultations in the first trimester of pregnancy; 5) Association measures used.

After reading the full article, the evaluation of the methodological quality of the included studies was carried out with the aid of the critical evaluation tool of cross-sectional studies for use in a systematic review by the Joanna Briggs Institute [8]. This tool consists of eight questions that assess the methodological quality of the study and determine the possibility of bias in its design, conduction, and analysis. Associated with this instrument, the score described by Taylor was used as a strategy to stratify the risk of bias [9]. In it, the studies are classified as “low risk of bias,” if a “yes” score ≥ of 70% is obtained; “Moderate risk of bias,” if the “yes” score is between 50% and 69%; and “high risk of bias,” if the “yes” score is less than 50% [10]. This assessment was carried out independently by the two reviewers and the disagreements were resolved by consensus.

The Review Manager software (RevMan; version 5.3.5–available at: (http://community.cochrane.org/tools/review-production-tools/revman-5/revman-5-download): was used for statistical analysis of results and construction of the meta-analysis graphs, using the random-effects model and Odds Ratio (OR) as a measure of association for dichotomous data, with a 95% confidence interval (CI).

We quantify the inconsistencies between the results of the studies using the I^2^ statistic, which illustrates the percentage of variability in the effect estimates that surpasses the effect of chance: I^2^ = [(Q–df)/Q] x 100%, where Q is the statistic Chi [2] and df the degree of freedom. We evaluated the heterogeneity between the studies with a visual examination of the meta-analysis graphs to verify overlapping of the confidence intervals, using the Chi [2] test for homogeneity with a significance level of 5% and considering the I^2^ statistic [8]. An I^2^ value of less than 50% corresponded to low heterogeneity; 50% or greater, significant heterogeneity and 75% or greater, substantial heterogeneity [8].

This study received support from the Coordination for the Improvement of Higher Education Personnel (CAPES) through payment of a master’s scholarship [001] and Federal University of Rio Grande do Norte.

## Results

**FIGURE 1:**
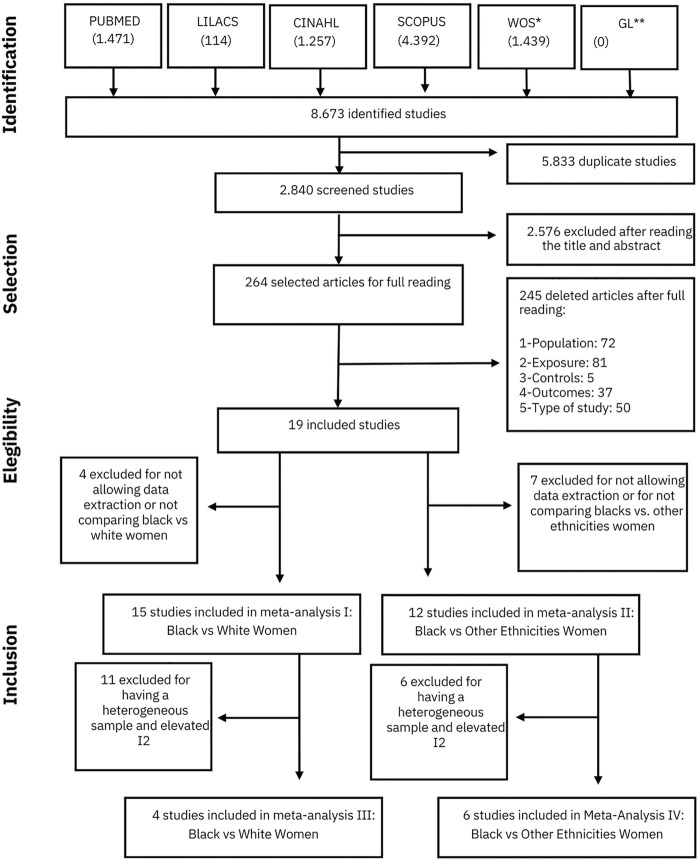
Flowchart for Article Selection Caption: * Web of Science; **Grey Literature Source: Designed by the authors (Brazil, 2021).

We identified 2,840 articles for screening by title and abstract. This evaluation resulted in 264 articles for full reading and 19 studies were then selected to compose this systematic review, being subjected to evaluation of the methodological quality and data extraction (Figure 1).

From the data obtained (Table 2), we found that the studies used the same gestational age (<12 weeks) to define the beginning of prenatal care. The population studied was described by ethnic groups (white, Asian, Hispanic, Latino), and also grouped into age group. For this characteristic, there is a great variety of presentations through age groups, and only one study [11] diverged, analyzing only pregnant adolescents.

**TABLE 2:** Characteristics of the included studies (*n* = 19) (Brazil, 2021).

Author, year	Place	Sample (n)	Age (years old)	Other ethnic-racial groups	Prevalence of access in the first trimester of pregnancy	Odds ratio (OR)
[14]	New York, United States	496	13–41	Hispanic	Black 59.1%	NR
					Hispanic 48.2%	
[17]	Texas, United States	533	Mean = 15,9 (SD = 1,1)	White, American Mexicans and Others	Blacks 47.8%	Black compared to American Mexican women (OR = 1.9 95% CI 1.2, 3.2) and white (OR = 1.7 95% CI 1.1, 3.0)
					White 55.8%	
					American Mexicans 59.3%	
					Another 33.3%	
[19]	New Jersey, United States	198.881	≤20, between 21 and 34 and above 35	White and black by geographic origin and country of birth	Black 62.7%	NR
					White 86.2%	
					Mexican black women 21.4%	
					Mexican whites 46.7%	
[20]	California, United States	3.071	>18	White, Hispanic (English speaking), Hispanic (Spanish speaking), Other	Black 12.3%	Black compared to white (OR = 1.33 95% CI 0.83–2.12
					English-speaking Hispanics 16.9%	
					Spanish-speaking Hispanics 36.6%	
					Other English-speaking ethnicities 5.6%	
					Other Spanish-speaking ethnicities 3.5%	
[21]	New Jersey, United States	91.585	≤15 to over 40	Non-Hispanic Whites, Hispanic Whites, and other Hispanics	Non-Hispanic Black Women 31.7%	Non-Hispanic black women compared to non-Hispanic women (OR = 0.65), other non-Hispanic women (OR = 0.63), Hispanic women (OR = 0.84 and other Hispanic women (OR = 0.84)
					Non-Hispanic Whites 44.9%	
					Hispanic Whites 36.5%	
					Other Hispanic 35.4%	
[16]	California, United States	6.364	≤20, 21–34, and ≥35	Latin, European, Asian Others	Afroamericanas 8.1%	African-American compared to Europeans (OR = 0.41 CI95% 0.20–0.86)
					Latin 64.8%	
					European 19.6%	
					Asian 7%	
					Other ethnicities 0.5%	
[22]	South Carolina, United States	7. 533	≤19 years old, 20–29 years old, and ≥30 years old	White	Use of the public system: Black 60%	Black women using the public health system are more likely to start prenatal care late when compared to black women (OR = 1.9, 95% CI 1.2–3.2) and white OR = 4.1 (95% CI 2.6–6.4) who had private health insurance
					White 73%	
					Use of private insurance	
					Black 85%	
					White 94%	
[23]	Washington, United States	766	12–17 years old, 18–25 years old, 26–34 years old, and ≥35 years old	White, Hispanic, and Other	NR	African-Americans having late prenatal care or not performing prenatal care vs. white women (OR = 2.3 95% CI 0.7–6.8)
[24]	South Carolina, United States	372	≤20 years old, 21–34 years old, and ≥35 years old	White	African American 64.3%	NR
					White 76.3%	
[25]	Florida, Georgia, New Jersey, Texas–United States	268.594	NR	Non-Hispanic White, Hispanic, and Asian/Pacific Islands	Non-Hispanic black women 72.3%	Black women vs. non-Hispanic white women in Georgia (OR = 0.75 CI95% 0.71–0.79) and Texas (OR = 0.73 CI95% 0.70–0.77)
					Non-Hispanic whites 87.9%	
					Hispanic 73.7%	
					Asian/Pacific Islands 82.1%	
[26]	Brazil and South Africa	Brazil: 3,761 women and 4,958 births South Africa: 4148 women and 4,800 births	15–49	Brazil: White South Africa: White and Asian	In Brazil	Brazil: no significant difference between white and mixed-race (OR = 0.93) and white and black (OR = 1.04). South Africa: less access to prenatal care among black women in the urban area (OR = 0.22) and black women in the rural area (OR = 0.22)
					Black 61.4%	
					Sparrow 61.6%	
					White 76.3%	
					In South Africa	
					Freckles 40.4%	
					White 77.5%	
					Asian 65.5%	
[8]	United States	4.219	10–40	Non-Hispanic Whites, Non-Hispanic Blacks, Hispanics, Asian/Pacific Islands, Native American/Alaska Native	NR	The 5% increase in the rate of black users reduces the rate of use of prenatal care in the first quarter by 1%. The 5% increase in white users increases the use of prenatal care in the first quarter by 1%
[27]	Georgia, United States	1.096	Between 18 and 34 years old	Non-Hispanic whites, Hispanics/others	Non-Hispanic Black Women 58,1%	Non-Hispanic blacks vs. non-Hispanic whites (OR = 0.44 95% CI 0.24–0.73)
					Non-Hispanic White Women 70,8%	
					Hispanic/others 81,8%	
[13]	Pelotas, Brazil	4244	Not reported	Whites	Black 60,1%	NR
					Sparrow 65,7%	
					White 77,9%	
[28]	Rio de Janeiro, Brazil	2.353	Mean = 24.6 years old	White and Yellow	Sparrow 73,6%	Brown vs. white (OR = 0.81 95% CI 0.70–0.93) and black vs. white (OR = 0.78 95% CI 0.58–1.05)
					Black 71,7%	
					White 77,8%	
[29]	United States	5.200	15–19; 20–24; 25–29; 30–34; ≥35	White and Hispanic	Black 74.81%	NR
					White 89.63%	
					Hispanic 78.56%	
[30]	United States	519	Mean = 31.8	White, Hispanic, or Latin	Black 23%	NR
					White 65.5%	
					Hispanic 15.6%	
[18]	United States	167.463	18–24; 25–29; 30–34; +35	Native Americans/Native Alaskans; Asians; Non-Hispanic whites; Hispanic	Non-Hispanic Black Women 85.9%	Non-Hispanic black women compared to white women (OR = 1.52; 95% CI 1.40–1.64) are more likely to start prenatal care late
					Native American/Native Alaskan 85.2%	
					Asian 84.9%	
					Non-Hispanic whites 87.7%	
					Hispanic 87.5%	
[31]	England, United Kingdom	122.275	<20; 20–24; 25–29; 30–34; 35–39; ≥40	British whites; Irish Whites; others white; others	African black women 79.1%	African black vs. white women (OR = 1.90 95% CI 1.80–2.00) are more likely to start prenatal care late
					Caribbean black women 70.3%	
					Other black women 66.7%	
					British whites 73.3	
					Irish whites 75.9%	

95% CI: 95% confidence interval; OR, odds ratio; SD, standard deviation; NR, not reported.

Most of the studies articles are from the United States of America; only two were Brazilian [12, 13].

In general, there is a significant difference between the racial groups studied in the included articles. Black women, when compared to other ethnicities, have a lower prevalence of early prenatal care in the first trimester, with prevalence ranging from 8.1% to 74.81%, while compared to white women, it varied from 44.9 to 94.0%.

After applying the analysis instrument for the risk of bias (Table 3), we observed that only four studies (21%) [14–18] have a moderate risk of bias, with the others considered low risk. Then, the selected articles have good methodological quality, adequately answer their research questions, allowing their results to be generalized.

**TABLE 3:** Methodological quality and bias risk analysis according to Joanna Briggs critical appraisal checklist for analytical cross-sectional studies (Brazil, 2021).

Item	1	2	3	4	5	6	7	8	9	10	11	12	13	14	15	16	17	18	19
Were the criteria for inclusion in the sample clearly defined?	+	+	+	+	+	+	+	+	−	+	−	+	+	-	+	+	+	+	+
Were the study subjects and the setting described in detail?	+	+	+	+	−	−	−	+	+	+	+	+	+	−	−	+	−	−	+
Was the exposure measured in a valid and reliable way?	+	+	+	+	+	+	+	+	+	−	−	−	+	+	+	+	+	+	+
Were objective, standard criteria used for measurement of the condition?	+	+	−	+	+	+	+	+	+	−	−	−	+	+	−	+	−	−	+
Were confounding factors identified?	+	+	+	+	+	+	+	+	+	+	+	+	+	+	+	+	+	+	+
Were strategies to deal with confounding factors stated?	+	+	+	+	+	+	+	+	+	+	+	+	+	+	+	+	+	+	+
Were the outcomes measured in a valid and reliable way?	+	+	−	−	+	+	+	+	−	−	−	−	+	+	+	−	−	−	−
Was appropriate statistical analysis used?	+	+	+	+	+	+	+	+	+	+	+	+	+	+	+	+	+	+	+
Bias risk	L	L	L	L	L	L	L	L	L	M	M	M	L	L	L	L	M	M	L

[8, 11–16, 18–27, 29, 31].

+: Low risk; −: High risk; L: Low Risk; M: Moderate Risk.

Only 15 (78.9%) of the 19 studies included in the systematic review were included in meta-analysis I (black women versus white women); and 11 (57.9%) entered the meta-analysis II (black women compared to women of other ethnicities, except white). This is because from the data presented, it was not possible to extract the number of black women or other ethnicities belonging to the sample, or they did not make a comparison between whites and blacks or between blacks and other ethnicities.

**FIGURE 2:**
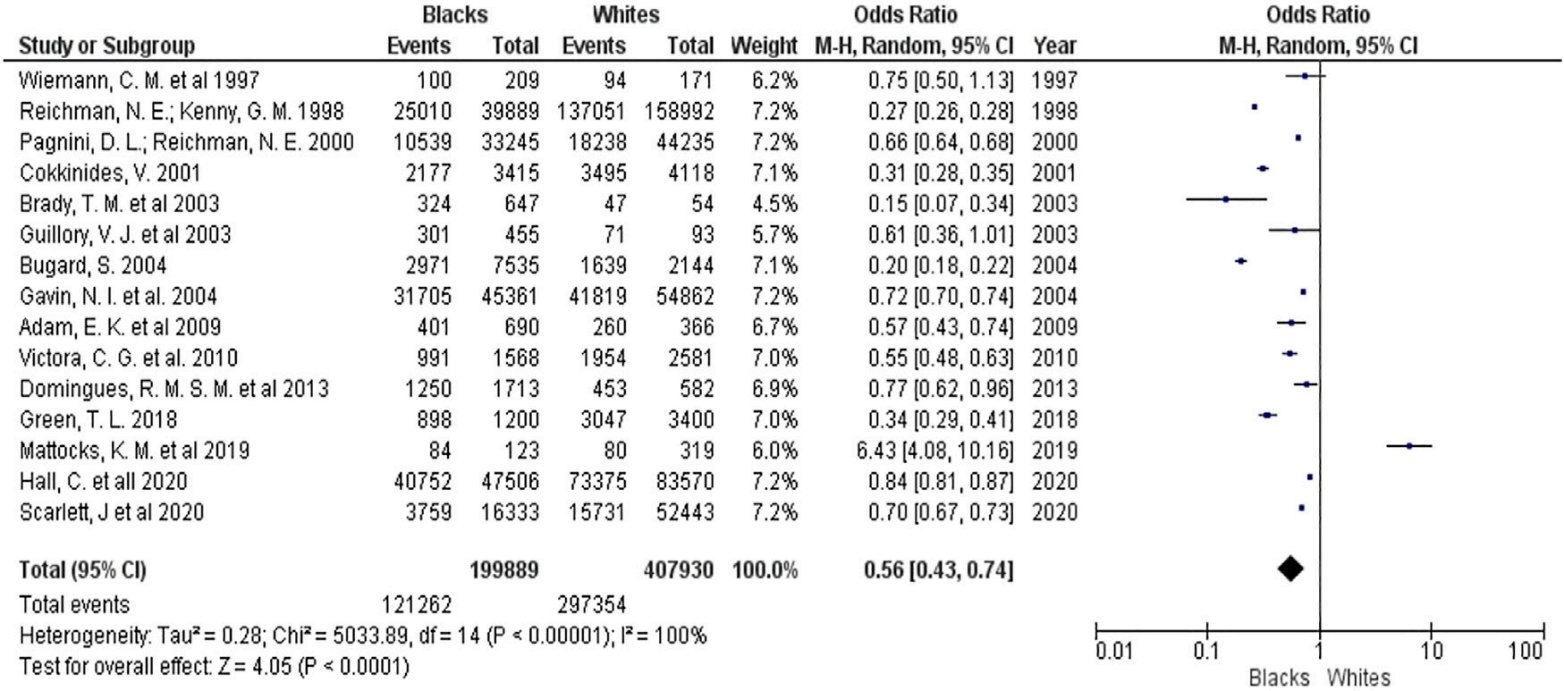
Forest Plot for the Early Start of Prenatal Care: Black women compared to white women (Brazil, 2021).

In meta-analysis I (Figure 2), we included 199,889 black women and 407,930 white women. We observed that 60.7% (95% CI = 60.5–60.9) of black women started prenatal care in the first trimester, while 72.9% (95% CI = 72.8–73.0) of white women did so. Black women are less likely to start their obstetric care early, with an OR of 0.56 (95% CI 0.43–0.74) compared to white women.

**FIGURE 3:**
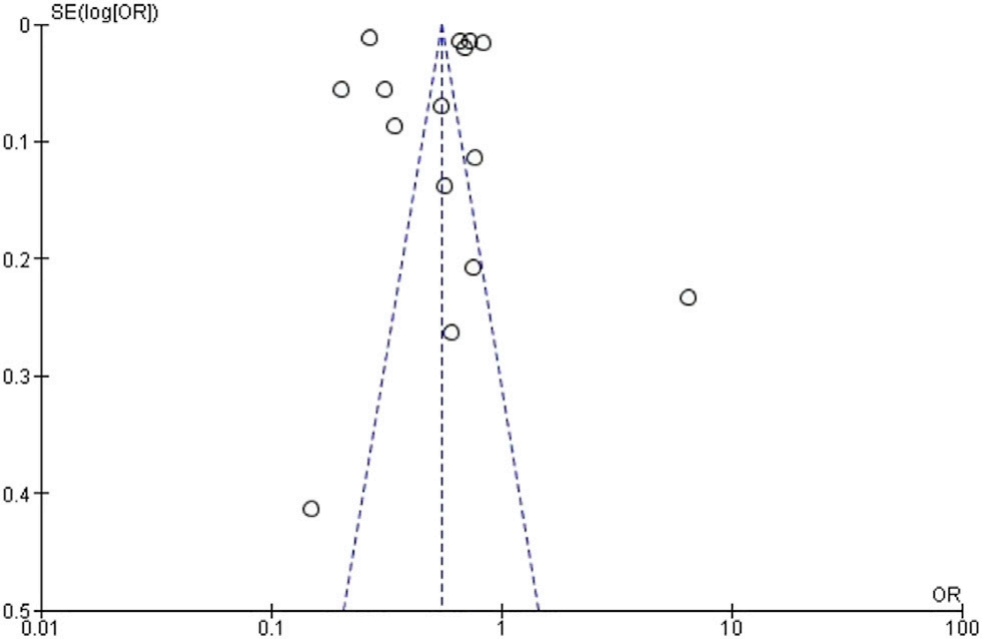
Funnel Plot for early prenatal care: black women compared to white women (Brazil, 2021).

However, the included studies are quite heterogeneous with I^2^ = 100% and the funnel plot (Figure 3) presents studies with varying data accuracy and outside the area covered by the plot, suggesting the presence of potential publication bias.

When comparing black women with women of other ethnicities (except white)–meta-analysis 2, we included 153,593 black women and 111,002 women of other ethnicities (Supplementary File S1), showing that the chances of black women starting prenatal care at the age of the first trimester are the same as women from other ethnic minorities (OR = 1.0; 95% CI 0.90–1.10) (Supplementary File S1).

Even so, we found a high heterogeneity (I^2^ = 91%) with the Funnel plot presenting studies with varying data accuracy and suggesting potential publication bias. (Supplementary File S2).

In an attempt to solve the high heterogeneity in the first two meta-analyses, two other meta-analyses were carried out grouping studies with methodologies and samples with similar characteristics (Supplementary File S3).

In this analysis, we studied 2,922 black women and 3,211 white women. We observed that 61.3% of black women start prenatal care in the first trimester, while for white women the prevalence was 74%. The result of the meta-analysis showed that the summary measure was OR = 0.57 (95% CI 0.51–0.64), which means that black women are 43% less likely to start prenatal care in the first trimester (Supplementary File S4), obtaining an I^2^ = 0% (Supplementary File S4).

In the following analysis, we compared 26,047 black women with 22,953 non-black women belonging to other ethnicities (excluding white ones), noting that black women have 16% less chance of gaining access to these services (OR = 0.84; 95% CI 0.71–0.99), with an I^2^ = 50% (Supplementary Files S5, S6).

## Discussion

This study aimed to analyze the prevalence of access to prenatal care during the first trimester of pregnancy by black pregnant women compared to white women and other ethnicities. As the results show, the prevalence of access to this service was lower among black women, varying from 8.1% to 74.81%, with fewer chances of accessing prenatal care than white women (OR = 0.57, 95% CI 0.51–0.64) and women of other ethnicities (OR = 0.84, 95% CI 0.71–0.99).

Another study showed that race and ethnicity are significant predictors for access to antenatal care [32]. White and Asian women, when compared to black or African American women, had significantly greater chances of punctuality in prenatal care (OR 1.59 and 1.87, respectively) and frequency of prenatal care (OR 1.89 and 2.44, respectively) [32] which shows inequality in access not only in comparison to white women but also to other minorities.

From the qualitative assessment of the studies included in this review, we observed that 16 studies are from the US, 2 from Brazil, and 1 from England. The US and Brazil are two multiracial nations, with a colonialist and slavery background, which allows us to reflect on the importance of this context on how racial discrimination was shaped.

In a study in the US, authors identified that preterm and very premature births, as well as delayed prenatal care, in addition to being reported more frequently by black women compared to white women, were also associated with geographical factors and sociodemographic. It was more frequent in black women living in the southern US and counties with a high rate of racial segregation [33]. This may explain the findings of this review, in which most of the studies were conducted in the states of Texas [11], California [16, 20], Carolina do South [22, 24, 27], and the study by Gavin et al [25] that analyzed data from both Florida, Georgia and Texas and New Jersey. It is noteworthy that the results presented may not be globally representative, but specific to the countries of the inserted studies.

The race/skin color item has been widely used in clinical and public health studies as a variable, especially in the US to assess social inequities in health outcomes and treatments [34]. In that country, a vast literature defines race as an important health predictor and demonstrates that blacks are at a disadvantage when compared to whites [26].

However, in Brazil, there are still few studies that associate race as a variable that defines social inequities, especially about women’s health [2], because the connotation that this condition has acquired throughout history differs from that adopted by North Americans. For a long time, it was related to the skin color phenotype and the characteristics of the individuals and not the ancestry, added to its absence as a question of filling in censuses and health notification/information systems [35].

Another review identified that in Brazilian scientific production, only 19 studies addressed institutional racism and the health of black women [36]. The scarcity of studies on the health of black women may be a consequence of epistemic racism [2, 36, 37]. Besides making difficult to advance and consolidate the debate regarding racial inequalities associated with socioeconomic factors in health outcomes, this facto also contributes to the perpetuation of institutional racism in Brazil.

Despite these differences, the associated analysis of studies from both countries is possible due to the presence of similarities between them. In addition, both nations have reported high levels of race-related discrimination in interpersonal settings and education and health institutions [26].

Most of the American articles included are with subjects belonging to States where the African-American population is higher such as Georgia, Florida, New Jersey, New York, California, and Texas. These studies are similar as they analyzed women who used the services of MEDICAID, Federal and State health insurance for the health of pregnant women, children, the older people over 65, people with physical and intellectual disabilities, and low-income adults. Brazilian studies, when compared to American studies, have a similar methodological design, as they approached women users of the Brazilian Unified Health System (SUS), comparing them in terms of race, income, education, parity, marital status, and age group.

In this context, one of the limitations observed in the studies included in this work is the way in which race was measured. In biomedical literature, there is a consensus that racial/ethnic categories are inaccurate and changeable, and that their measurement in census and health data varies over time [26].

All included studies were submitted to methodological quality assessment and 14 studies had a low risk of bias, which shows the good methodological quality of the included studies. The main limitations found in most studies were related to failures in filling the item race or skin color in health records in some countries, which can generate information bias. In studies carried out in US, data on skin color came from databases of vital statistics and questionnaires sent electronically to participants, in which the fields referring to color/race were self-reported. In addition, the population classified as Latin was not stratified as white Latinos or black Latinos. In Brazilian studies, this variable was measured both by the participants’ self-declaration and by the interviewer’s judgment.

Also, the findings found here are the results of an analysis of observational studies of cross-sectional design, constituting one of the limitations of this review. The inclusion of only articles of this nature can lead to generalized conclusions since the confounding factors are not always adjusted properly, as they are analyzed at the same time. The lack of identification of confounding factors and the strategies to deal with these factors, mainly in studies carried out in the 1990s and early 2000s, were seen from the assessment of the risk of bias.

The high heterogeneity found in the first two meta-analyses carried out with all eligible studies and comparing black and white women; and black women and other ethnicities (except white), can be explained by an overestimated measure of association, based on studies with varying sample sizes, studies that included women with access to private services, who analyzed drug users and that focused only on teenagers.

Considering this and as a strategy to try to minimize the high heterogeneity obtained, we carried out two other meta-analyses, combining studies with participants with similar sociodemographic characteristics. Therefore, results were obtained with low heterogeneity and low publication bias, in addition to high data accuracy. Given these findings, we can assume that the difficulty in accessing black women to prenatal care is not, in fact, only related to socioeconomic status, and maybe the consequence of a much broader and more complex process such as racism.

Even when evaluated together with white women or other ethnicities with similar sociodemographic characteristics and being users of public health services, black women still have little or no participation in prenatal services [38, 39].

Black women are not carefully monitored like white women and, when they have symptoms, most of the time, they are not followed up with due attention [40]. Differences like these occur well before the patient arrives at the place of care and extend through direct care or gaps in communication between health professionals. Among other factors, the explanation for this phenomenon lies in the stereotype formed about people of black color and in racism, affecting the way they are treated in health services [40].

Racism consists of a system of oppression that gives value to people, based on race or ethnicity. There are categories of racism that can contribute to the increase in racial health disparities: 1) institutional racism; 2) personal racism; and 3) internalized racism. Everyone plays an important role in the negative experiences lived by black women during their sexual and reproductive trajectory [41].

Institutional racism is characterized by practices imposed by health organizations that negatively affect user access to the most diverse levels of care, resulting in differences in the quality of service offered to minority racial groups. This category of discrimination promotes attitudes, practices, beliefs, and policies that give advantages to whites and disadvantages to other ethnic groups [41].

Although government policies are created in an attempt to generate social changes that lead to equity of access [42], institutional racism is considered the source of social disparities in health, affecting treatment, the quality of care provided, extending to opportunities for housing, education, and employment [40].

Within these institutions with a discriminatory context, service users can also experience personal racism when the preconceived judgment of health professionals about certain racial groups, resulting in care below the standard provided for ethnic/racial majorities [43].

This type of behavior is seen in the stereotyped attitudes of doctors during their treatment recommendations for black patients, who are seen as promiscuous, receiving inferior and deficient treatments and delays in the screening inherent to prenatal care as rapid tests for the detection of syphilis and HIV in pregnancy, Pap smear exam, Pap smear, performed to track HPV-related pathologies [44, 45].

Finally, considered as a consequence of the previous two levels of discrimination, internalized racism refers to the acceptance and personification of society’s stigmatizing messages and attitudes by the oppressed racial groups. The internalization of these concepts has a major impact on the sexual and reproductive health of these women, causing psychological stress, use of illicit substances, and multimorbidities (gestational diabetes mellitus, gestational hypertension, premature birth, low birth weight), in addition to delaying the search for care, due to the fear, even if unconscious, of experiencing institutional and personal racism [41].

Given the above, studies that make associations between racism and the health of the black population are fundamental as thematic fields of research to observe their repercussions, impact, and ways of coping. Understanding the impact of racism on the sexual and reproductive health of women of African descent provides the context for analyzing the results obtained. The historical and contemporary health experiences of black women provide a perspective that can be considered by health professionals and others who offer services and implement programs, aiming at greater equity for these women.

## Conclusion

Black women have a lower prevalence of participation in prenatal care in the first trimester, ranging from 8.1% to 74.81%, with lower chances of accessing prenatal care when compared to white women and women of other ethnicities. The contribution of this study is to raise the discussion about the impact that racial inequity has on the health of individuals, encourage new studies that include the question of race or skin color in their analyzes, in addition to highlighting the need for adjustments between different ethnic-racial classifications.

The study results reveal a current and emerging theme to be worked on globally. Structural racism is present within health services, this practice contributes to the worst health indicators of the black population, as evidenced in this review. It is necessary to turn the attention of public health care policies to this population, in order to recover the long historical process of existing racism, so that all women are treated equally, in addition to working on changes in professional practice, encouraging behavioral changes that reflect on maternal health care.

## Supplementary Material

Supplementary File S1 Forest Plot Early prenatal care: black women compared to women of other 517 ethnicities (except white) (Brazil, 2021).

Supplementary File S2 Funnel Plot for Early onset of prenatal care: black women compared to 520 women of other ethnicities (except white) (Brazil, 2021).

Supplementary File S3 Forest Plot Early onset of prenatal care: black women compared to white 523 women (Brazil, 2021).

Supplementary File S4 Funnel Plot Early onset of prenatal care: black women compared to white 526 women (Brazil, 2021).

Supplementary File S5 Forest Plot Early Start of Prenatal Care: Blacks compared to Other 529 Ethnicities (Brazil, 2021).

Supplementary File S6 Funnel Plot Early onset of prenatal care: black women compared to other 532 ethnicities (Brazil, 2021).
