# Supplementary figures and images for: Prevalence of Access to Prenatal Care in the First Trimester of Pregnancy Among Black Women Compared to Other Races/Ethnicities: A Systematic Review and Meta-Analysis
Source: Public Health Rev. 2022 Jul 4;43:1604400. doi: 10.3389/phrs.2022.1604400 (PMC9289875; doi:10.3389/phrs.2022.1604400)

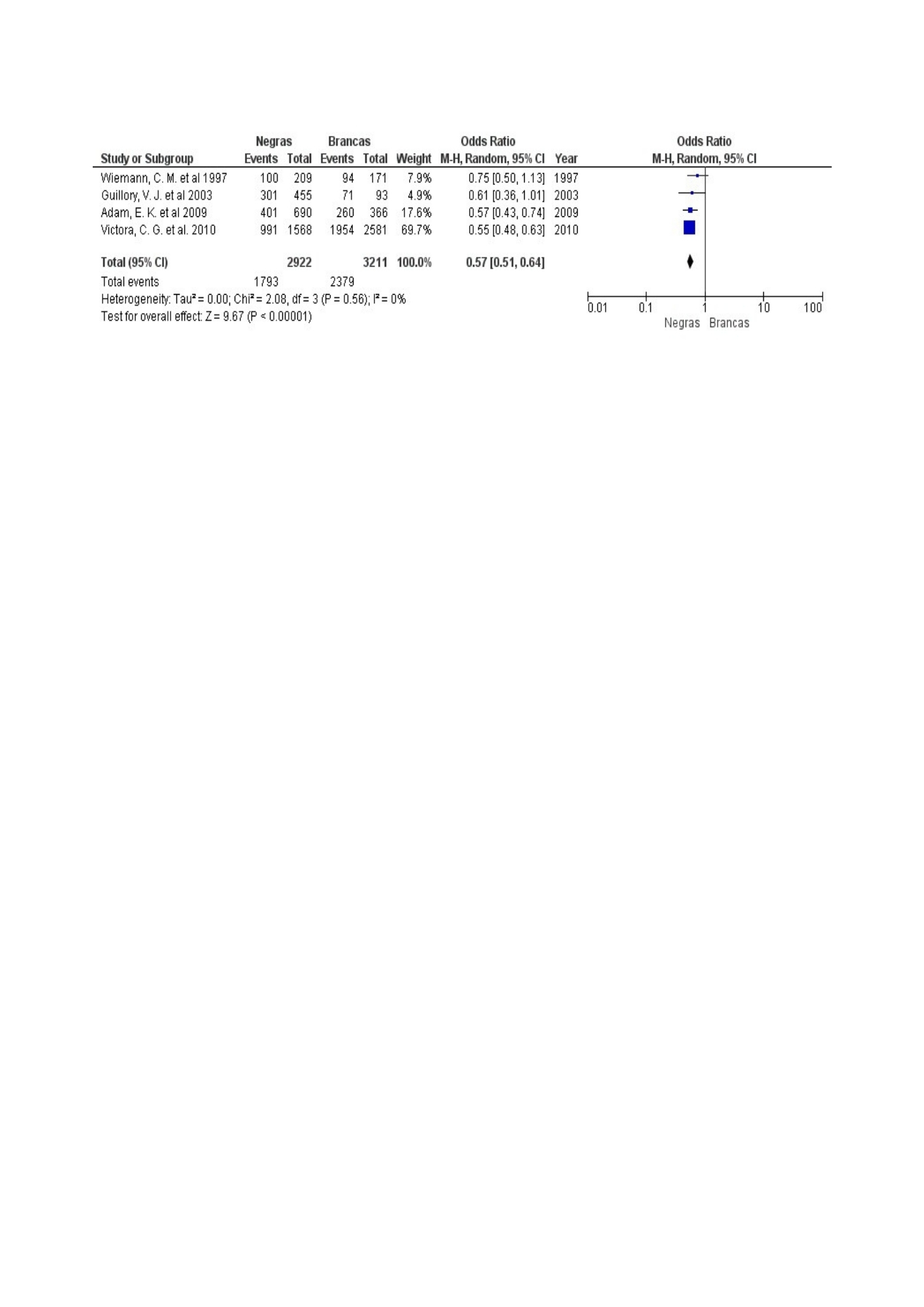

Supplement: Supplementary file 1 [file Image3.JPEG]

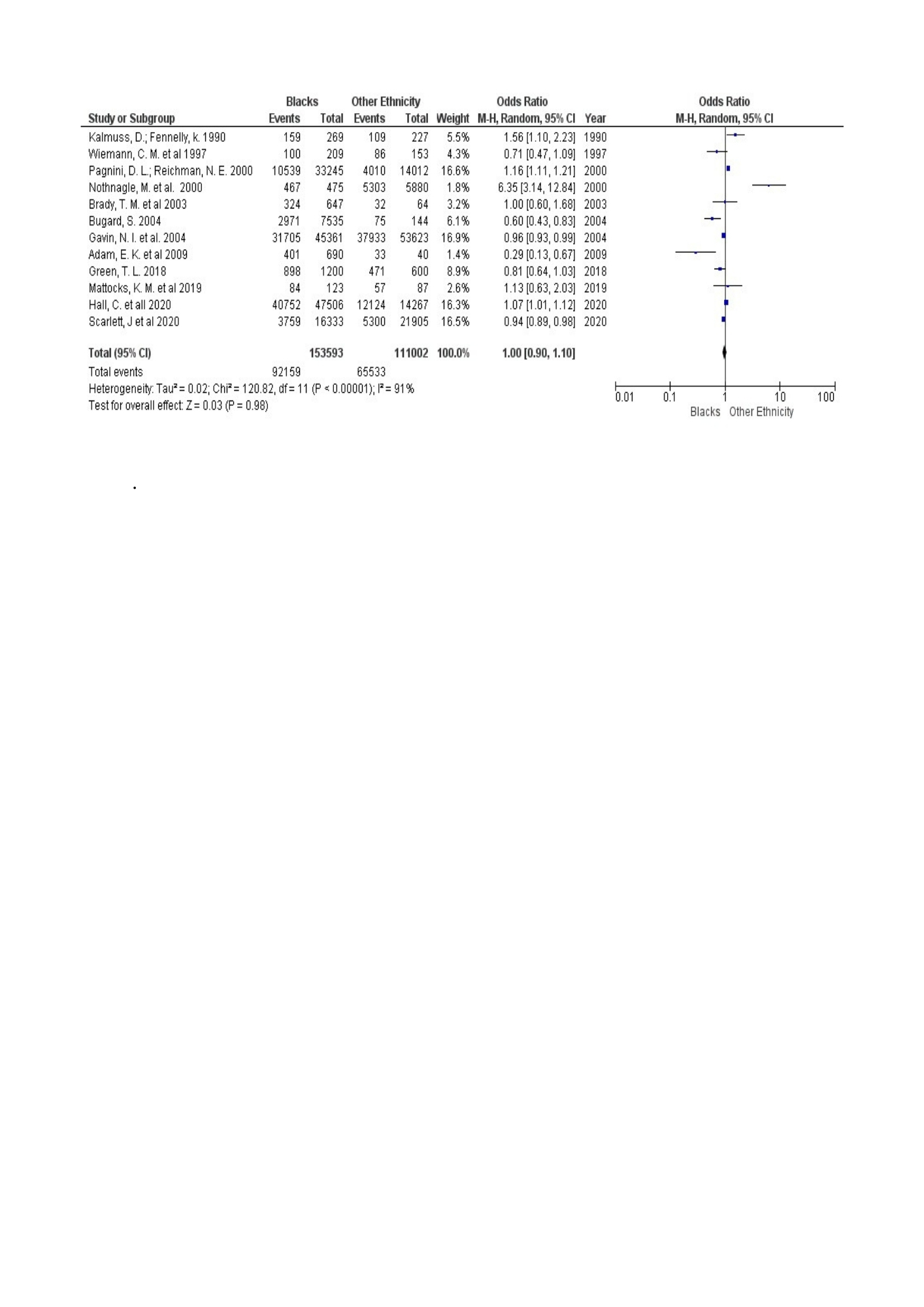

Supplement: Supplementary file 2 [file Image1.JPEG]

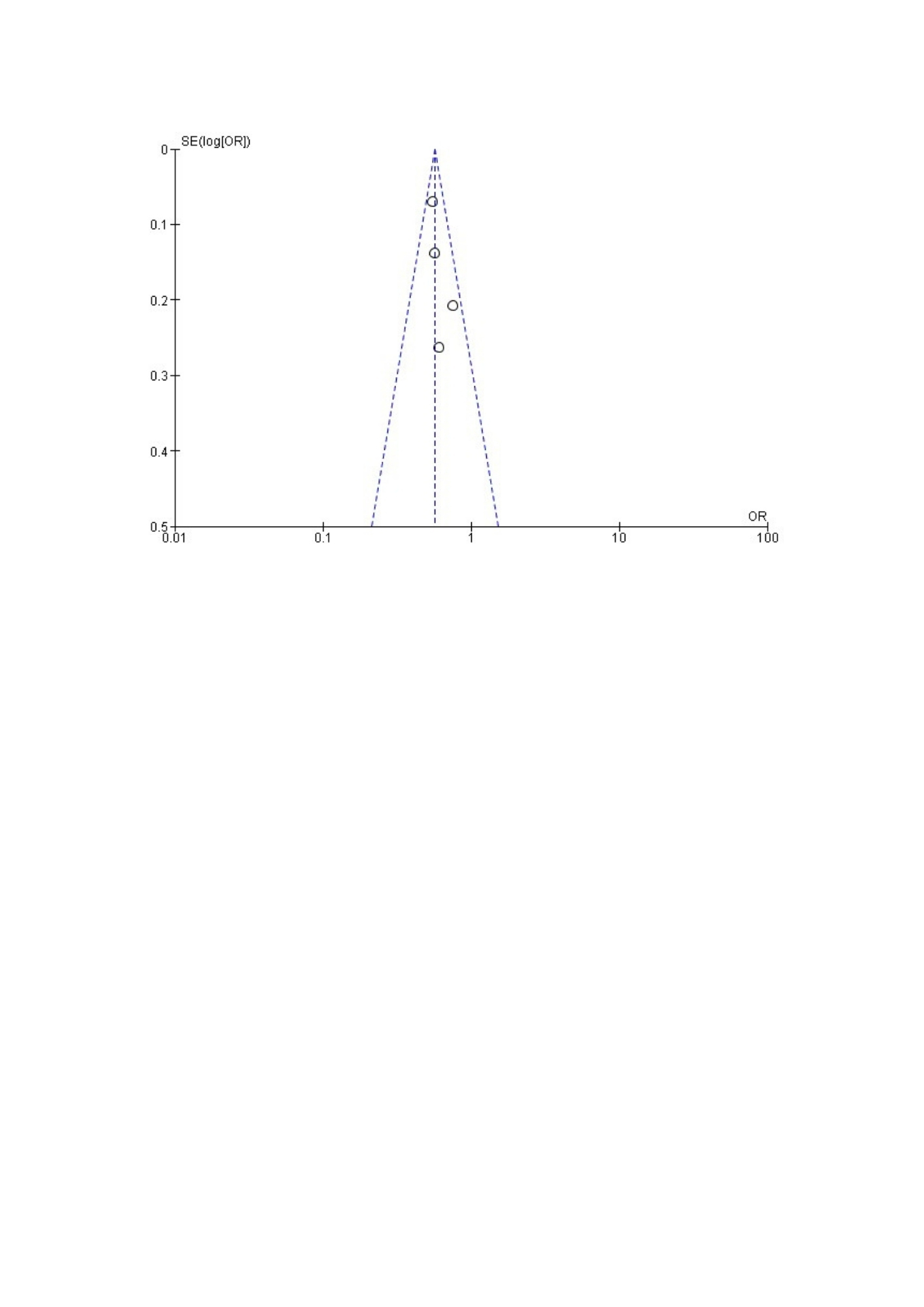

Supplement: Supplementary file 3 [file Image4.JPEG]

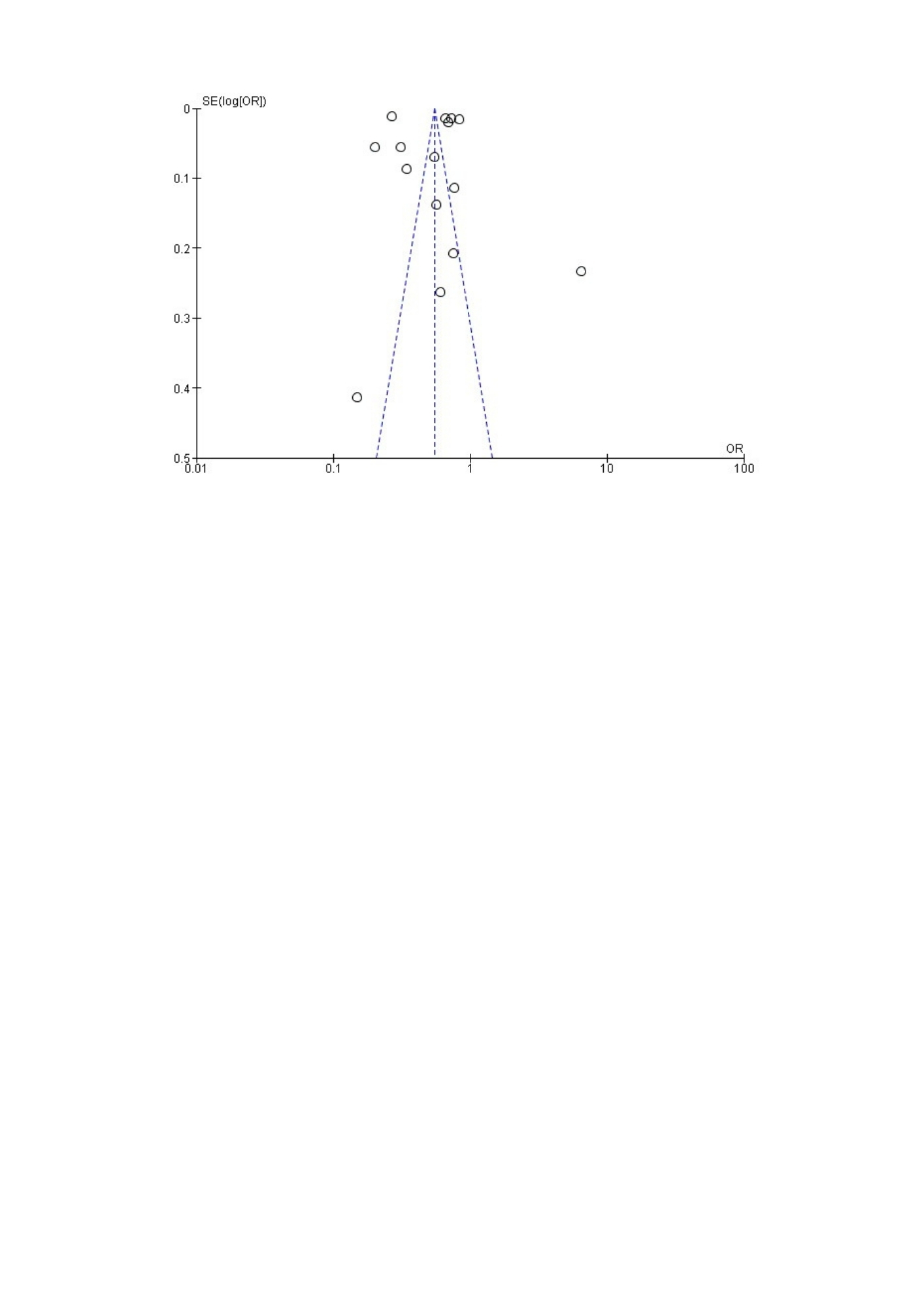

Supplement: Supplementary file 4 [file Image2.JPEG]

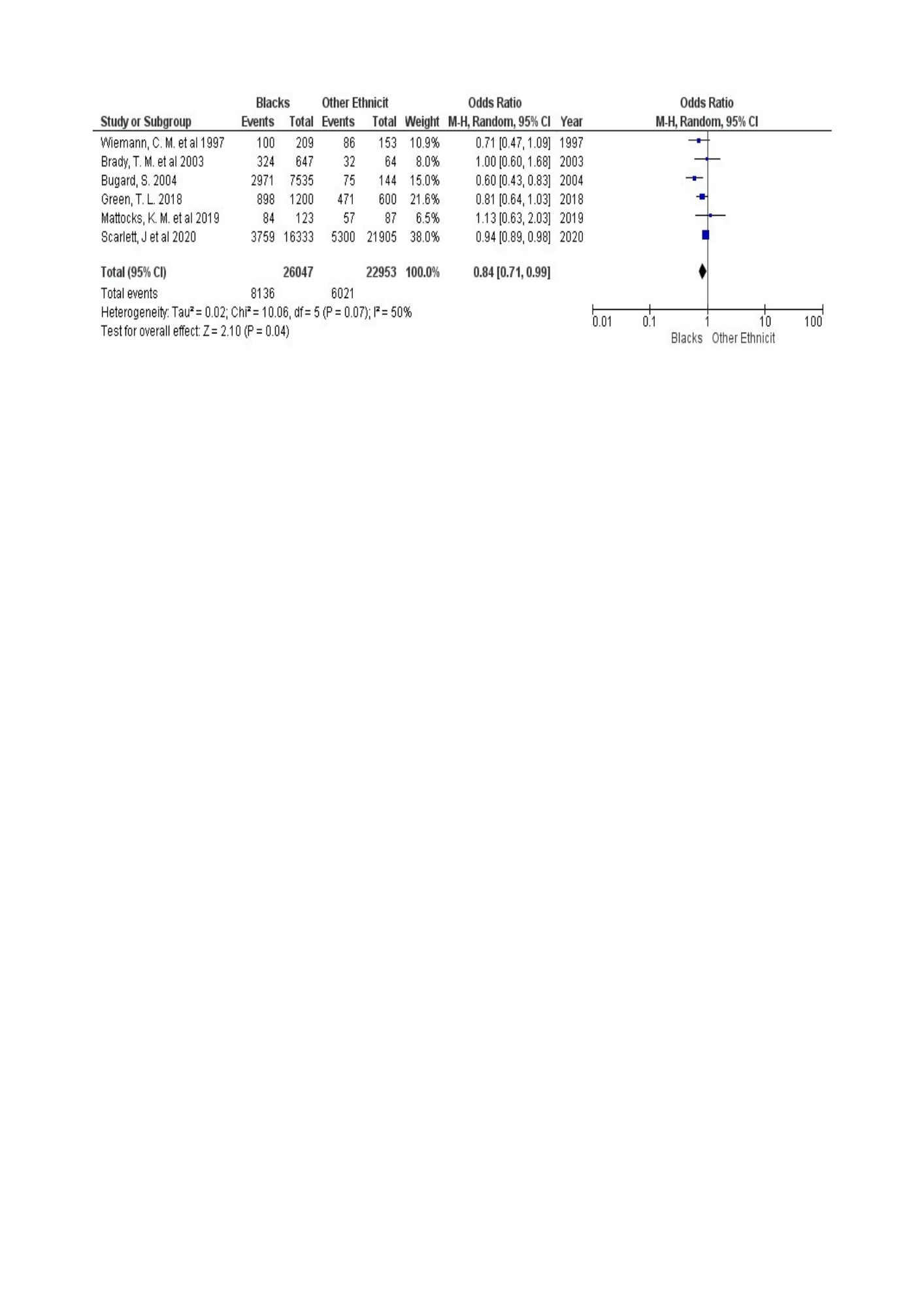

Supplement: Supplementary file 5 [file Image5.JPEG]

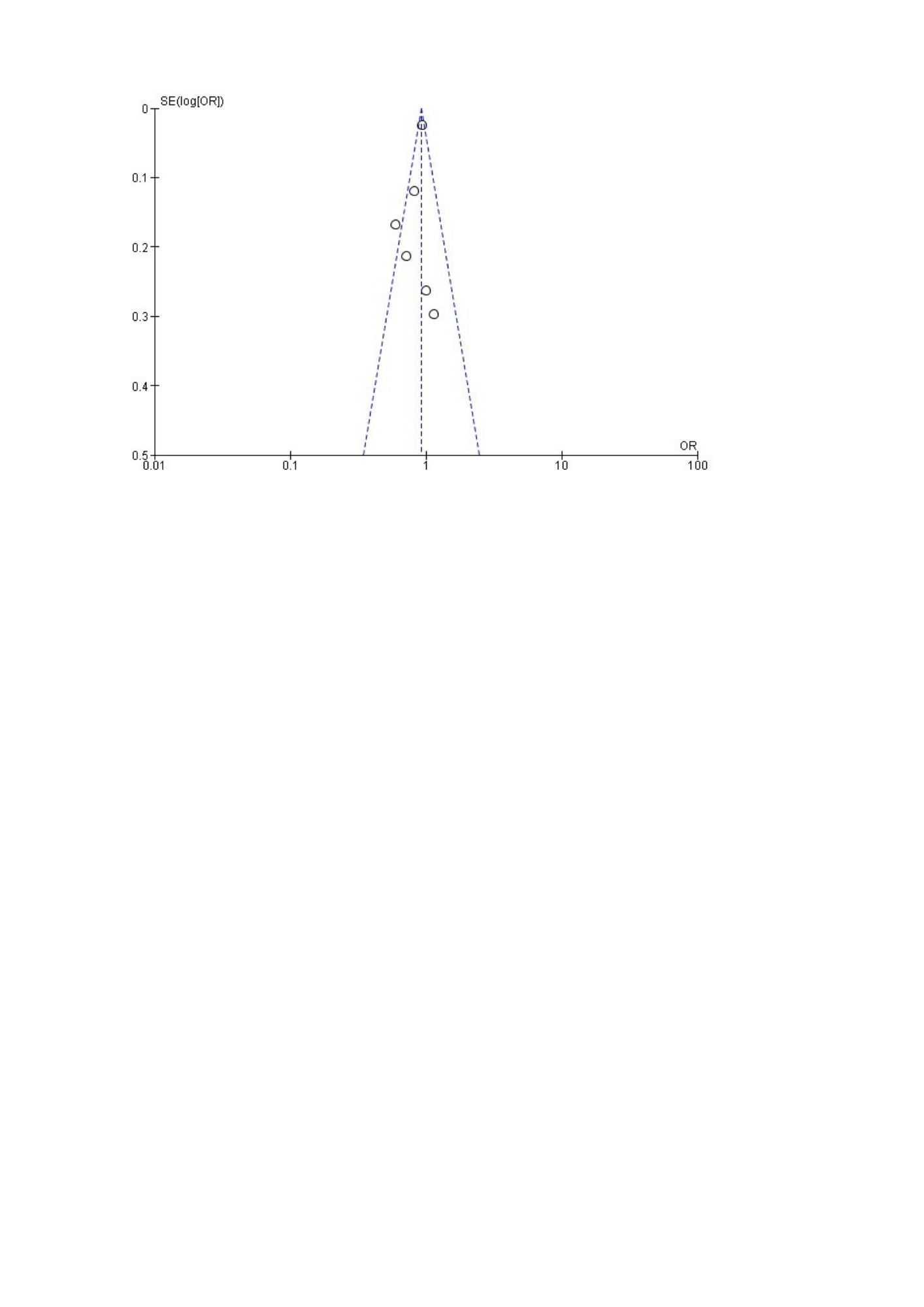

Supplement: Supplementary file 6 [file Image6.JPEG]
